# Expanding the range of polyhydroxyalkanoates synthesized by methanotrophic bacteria through the utilization of omega-hydroxyalkanoate co-substrates

**DOI:** 10.1186/s13568-017-0417-y

**Published:** 2017-06-05

**Authors:** Jaewook Myung, James C. A. Flanagan, Robert M. Waymouth, Craig S. Criddle

**Affiliations:** 1 Department of Civil and Environmental Engineering, Stanford University, Stanford, CA 94305 USA; 2 Department of Chemistry, Stanford University, Stanford, CA 94305 USA; 3 Woods Institute for the Environment, Stanford, CA 94305 USA; 4 William and Cloy Codiga Resource Recovery Center, Stanford, CA 94305 USA; 5 Department of Civil and Environmental Engineering, Pennsylvania State University, University Park, PA 16802 USA; 6 Brighton College, Eastern Road, Brighton, BN2 0AL UK

**Keywords:** Methane, Methanotroph, PHA, Biopolymer, Copolymer, Omega hydroxy acids

## Abstract

The first methanotrophic syntheses of polyhydroxyalkanoates (PHAs) that contain repeating units beyond 3-hydroxybutyrate and 3-hydroxyvalerate are reported. New PHAs synthesized by methanotrophs include poly(3-hydroxybutyrate-*co*-4-hydroxybutyrate) (P(3HB-*co*-4HB)), poly(3-hydroxybutyrate-*co*-5-hydroxyvalerate-*co*-3-hydroxyvalerate) (P(3HB-*co*-5HV-*co*-3HV)), and poly(3-hydroxybutyrate-*co*-6-hydroxyhexanoate-*co*-4-hydroxybutyrate) (P(3HB-*co*-6HHx-*co*-4HB)). This was achieved from a pure culture of *Methylocystis parvus* OBBP where the primary substrate is methane and the corresponding ω-hydroxyalkanoate monomers are added as a co-substrate after the cells are subjected to nitrogen-limited conditions.

**Electronic supplementary material:**

The online version of this article (doi:10.1186/s13568-017-0417-y) contains supplementary material, which is available to authorized users.

## Introduction

Polyhydroxyalkanoates (PHAs) are microbial storage polymers accumulated by many different groups of bacteria as an intracellular carbon and energy reserve. PHAs are biodegradable, biocompatible, and renewable bioplastics (Myung et al. 2014) that could substitute for petrochemical-derived plastics in many applications. Accumulation of PHAs occurs when bacterial cells grow under conditions where substrates other than the electron donor (typically the carbon source), such as nitrogen or phosphorus, limit growth. Depending upon the carbon co-substrates supplied during this nutrient-limited period, PHAs with different compositions are produced. Over one hundred different carboxylic acid monomers are reported to be incorporated into PHAs, resulting in polymers with a wide range of material properties (Steinbüchel and Gorenflo 1997).

Among the variety of polymers produced, 4-hydroxybutyrate (4HB) homopolymer or its copolymer are of interest for various biomedical applications (Martin and Williams 2003). It is a strong, flexible thermoplastic material that can be processed easily to form scaffolds, heart valves, or cardiovascular tissue supports (Martin and Williams 2003). In addition, 4HB polymer is extremely well tolerated in vivo because biological hydrolysis of 4HB homopolymer or copolymer yields 4HB, which is a common metabolite in the human body (Nelson et al. 1981). A copolymer of 3-hydroxybutyrate (3HB) and 4HB units is degradable by lipase and PHA depolymerase, in contrast to most PHAs, which cannot be degraded by lipase (Saito and Doi 1994; Wu et al. 2009). Aside from 4HB, the presence of structurally similar monomer units such as 5-hydroxyvalerate (5HV) and 6-hydroxyhexanoate (6HHx) in PHAs also adds elasticity to the polymer and enhances lipase-mediated degradation of the polymer (Mukai et al. 1993).

Use of methane (CH_4_) as a feedstock for PHA production can significantly decrease costs and environmental impacts (Rostkowski et al. 2012; Strong et al. 2015). Methane is currently widely available as the major component of natural gas and biogas obtained from the anaerobic degradation of organic waste. When CH_4_ is the sole feedstock, high molecular weight poly(3-hydroxybutyrate) (P3HB) is the sole PHA product (Wendlandt et al. 2001; Pfluger et al. 2011; Pieja et al. 2011; Myung et al. 2015b, 2016b). Recently, we reported production of poly(3-hydroxybutyrate-*co*-3-hydroxyvalerate) (PHBV) copolymer by a methanotrophic enrichment (Myung et al. 2015a) and a pure culture of obligate Type II methanotrophs (Myung et al. 2016a) when fed CH_4_ as a primary feedstock and valerate as a co-substrate.

In general, bacterial enzymes involved in PHA synthesis have broad substrate specificity (Poirier et al. 1995). For example, in *Alcaligenes eutrophus*, the PHA synthase can incorporate 3-hydroxyvalerate (3HV), 4HB, 4-hydroxyvalerate, 5HV, and 4-hydroxyhexanoate into PHAs (Haywood et al. 1989; Valentin et al. 1992, 1994). To our knowledge, this same broad specificity for substrates was not known for methanotrophic bacteria. Herein, we report the first methanotrophic synthesis of PHAs that contain repeating units beyond 3HB and 3HV, including poly(3-hydroxybutyrate-*co*-4-hydroxybutyrate) (P(3HB-*co*-4HB)), poly(3-hydroxybutyrate-*co*-5-hydroxyvalerate-*co*-3-hydroxyvalerate) (P(3HB-*co*-5HV-*co*-3HV)), and poly(3-hydroxybutyrate-*co*-6-hydroxyhexanoate-*co*-4-hydroxybutyrate) (P(3HB-*co*-6HHx-*co*-4HB)). This was achieved by a pure culture of *Methylocystis parvus* OBBP when the primary substrate is CH_4_ and the corresponding ω-hydroxyalkanoate monomers are added as co-substrates.

## Materials and methods

### Culture conditions

Unless otherwise specified, all *Methylocystis parvus* OBBP cultures were grown in medium JM2, which is a modified version of ammonium mineral salts (AMS) medium (Whittenbury et al. 1970). Medium JM2 contained the following chemicals per L of solution: 2.4 mM MgSO_4_·7H_2_O, 0.26 mM CaCl_2_, 36 mM NaHCO_3_, 4.8 mM KH_2_PO_4_, 6.8 mM K_2_HPO_4_, 10.5 μM Na_2_MoO_4_·2H_2_O, 7 μM CuSO_4_·5H_2_O, 200 μM Fe-EDTA, 530 μM Ca-EDTA, 5 mL trace metal solution, and 20 mL vitamin solution. The trace stock solution contained the following chemicals per L of solution: 500 mg FeSO_4_·7H_2_O, 400 mg ZnSO_4_·7H_2_O, 20 mg MnCl_2_·7H_2_O, 50 mg CoCl_2_·6H_2_O, 10 mg NiCl_2_·6H_2_O, 15 mg H_3_BO_3_ and 250 mg EDTA. The vitamin stock solution contained the following chemicals per L of solution: 2.0 mg biotin, 2.0 mg folic acid, 5.0 mg thiamine·HCl, 5.0 mg calcium pantothenate, 0.1 mg vitamin B12, 5.0 mg riboflavin and 5.0 mg nicotinamide.

All cultures were incubated in 160 mL serum bottles (Wheaton, Millville, NJ, USA) capped with butyl-rubber stoppers and crimp-sealed under a CH_4_:O_2_ headspace (molar ratio 1:1.5; >99% purity; Praxair Technology, Inc., Danbury, CT, USA). The liquid volume was 50 mL, and the headspace volume was 110 mL. Cultures were incubated horizontally on orbital shaker tables at 150 rpm. The incubation temperature was 30 °C.

### Synthesis of ω-hydroxyalkanoate monomers

The general preparation is a modification of a literature method (Takashima et al. 2004): a 20 mL vial was charged with 6 mL of a 4 M aqueous solution of sodium hydroxide. 2 g of the lactone was slowly added, and the contents stirred for 24 h. Volatiles were then evaporated, and the residue washed. The resulting white powders were dried overnight.

#### *4*-*Hydroxybutanoate (4*-*hydroxybutyrate)* (from γ-butyrolactone). 78% yield

^1^H-NMR (Additional file 1: Figure S1a, 500 MHz, D_2_O) δ 3.54 (t, *J* = 7.8, 2H), 2.18 (t, *J* = 8.6, 2H), 1.69–1.60 (m, 2H). ^13^C-NMR (Additional file 1: Figure S1b, 125 MHz, D_2_O) δ 183.7, 62.1, 34.6, 29.0. This is in agreement with the literature (Rival et al. 2012).

#### *5*-*Hydroxypentanoate (5*-*hydroxyvalerate)* (from δ-valerolactone) 72% yield

^1^H-NMR (Additional file 1: Figure S2a, 500 MHz, DMSO-d_6_) δ 3.33 (t, *J* = 6.4 Hz, 2H), 1.88 (dd, *J* = 8.3, 5.9 Hz, 2H), 1.53–1.29 (m, 4H). ^13^C-NMR (Additional file 1: Figure S2b, 125 MHz, DMSO-d_6_) δ 177.8, 60.5, 38.0, 33.9, 22.6. This is in agreement with the literature (Takashima et al. 2004).

#### *6*-*Hydroxyhexanoate* (from ε-caprolactone) 74% yield

^1^H-NMR (Additional file 1: Figure S3a, 500 MHz, D_2_O) δ 3.55 (t, *J* = 6.6 Hz, 2H), 2.14 (t, *J* = 7.4 Hz, 2H), 1.57–1.46 (m, 4H), 1.32–1.25 (m, 2H). ^13^C-NMR (Additional file 1: Figure S3b, 125 MHz, D_2_O) δ 184.0, 61.6, 37.5, 31.0, 25.6, 24.9. This is in agreement with the literature (Lemoine et al. 2014).

Alternatively, the sodium ω-hydroxyalkanoate monomers can be prepared in situ by dissolving 1 g of lactone in approximately 2.5 mL of 4 M aqueous sodium hydroxide and adjusting the pH to 7.

### Balanced growth phase and unbalanced PHA production phase

Fifty-milliliter *Methylocystis parvus* OBBP cultures were grown to final optical densities (OD_600_) of 0.8–1.2 then centrifuged (3000*g*) for 15 min. The pellets were resuspended in 30 mL of JM2 medium to create the inoculum for triplicate 160 mL serum bottle cultures. Each culture received 10 mL inoculum plus 40 mL of fresh medium (39.5 mL of medium JM2 plus 0.5 mL of 1.35 M ammonium chloride stock) and was flushed for 5 min with a CH_4_/O_2_ mixture (molar ratio of 1:1.5). After growth at 30 °C for 24 h, the headspace in each culture was again flushed for 5 min with the CH_4_/O_2_ mixture then incubated at 30 °C for a second 24 h period of balanced growth.

After 48 h, all cultures were harvested and subjected to nitrogen-limiting conditions. Triplicate samples were centrifuged (3000*g*) for 15 min and suspended in fresh medium without nitrogen. The headspace of each bottle was flushed with the CH_4_:O_2_ gas mixture at t = 0 h and t = 24 h. To assess the effects of co-substrate addition of PHA synthesis, the medium was amended with varying concentrations of 4HB, 5HV, and 6HHx monomers. Other organic acid co-substrates including 3HB, butyrate, valerate, hexanoate, and octanoate (Sigma-Aldrich, St Louis, MO, USA) were also tested for PHA copolymer synthesis. After 48 h of incubation, cells were harvested by centrifugation (3000*g*) and freeze-dried. Preserved samples were assayed for PHA content.

### PHA weight percentages

To determine PHA weight percent, between 5 and 10 mg of freeze-dried biomass were weighed then transferred to 12 mL glass vials. Each vial was amended with 2 mL of methanol containing sulfuric acid (3%, vol/vol) and benzoic acid (0.25 mg/mL methanol), supplemented with 2 mL of chloroform, and sealed with a Teflon-lined plastic cap. All vials were shaken and then heated at 95–100 °C for 3.5 h. After cooling to room temperature, 1 mL of deionized water was added to create an aqueous phase separated from the chloroform organic phase. This was mixed on a vortex mixer for 30 s then allowed to partition until phase separation was complete. The organic phase was aspirated by syringe and analyzed using a gas chromatograph (Agilent 6890N; Agilent Technologies, Palo Alto, CA, USA) equipped with an HP-5 column (containing (5% phenyl)-methylpolysiloxane; Agilent Technologies, Palo Alto, CA, USA) and a flame ionization detector. dl-3-Hydroxybutyric acid sodium salt (Sigma-Aldrich, St Louis, MO, USA) and was used to prepare external calibration curves. The PHA content (wt%, w_P3HB_/w_CDW_) of the samples were calculated by normalizing to initial dry mass.

### Analytical methods

To analyze concentrations of CH_4_ and O_2_, 0.5 mL of gas phase from each enrichment culture was injected onto a GOW-MAC gas chromatograph with an Alltech CTR 1 column and a thermal conductivity detector. The following method parameters were used: injector, 120 °C; column, 60 °C; detector, 120 °C; and current, 150 mV. Peak areas of CH_4_ and O_2_ were compared to standards and quantified using the software ChromPerfect (Justice Laboratory Software, Denville, NJ, USA).

Concentrations of organic acids were determined using a Dionex DX-500 ion chromatograph (Dionex, Sunnyvale, CA, USA) equipped with a GP50 gradient pump, CD25 conductivity detector, AS40 Automated Sampler, an AS11-HC ion-exchange column, and eluted with a mobile phase containing sodium hydroxide using Chromeleon software (Dionex, Sunnyvale, CA, USA). Organic acids were qualified and quantified using pure standards (HPLC grade).

To analyze total suspended solids (TSS), 0.5–5.0 mL of cell suspension was filtered through pre-washed, dried, and pre-weighed 0.2 μm membrane filters (Pall, Port Washington, NY, USA). The filtered cells and membrane filters were dried at 105 °C for 24 h, then weighed on an AD-6 autobalance (Perkin Elmer, Norwalk, CT, USA).

### Material characterization

#### Purification

PHA granules were extracted from the cells by suspending 500 mg of freeze-dried cell material in 50 mL Milli-Q water, adding 400 mg of sodium dodecyl sulfate (>99.0% purity; Sigma-Aldrich, St. Louis, MO, USA) and 360 mg of EDTA, followed by heating to 60 °C for 60 min to induce cell lysis. The solution was centrifuged (3000*g*) for 15 min, and the pellet washed three times with deionized water. To purify the PHA, pellets were washed with a 50 mL sodium hypochlorite (bleach) solution (Clorox 6.15%), incubated at 30 °C with continuous stirring for 60 min, then centrifuged (3000*g*) for 15 min. Sample pellets were washed and re-centrifuged three times with deionized water.

#### Molecular weight

Molecular weights of PHAs were evaluated using gel permeation chromatography (GPC). Sample pellets were dissolved in chloroform at a concentration of 5 mg/mL for 90 min at 60 °C,  filtered through a 0.2 μm PTFE filter, and then analyzed with a Shimadzu UFLC system (Shimadzu Scientific Instruments, Columbia, MD, USA) equipped with a Shimadzu RID-10A refraction index detector. The GPC was equipped with a Jordi Gel DVB guard column (500 Å, Jordi Labs, Mansfield, MA, USA) and Jordi Gel DVB analytical columns (10^5^ Å, Jordi Labs, Mansfield, MA, USA). The temperature of the columns was maintained at 40 °C, and the flow rate of the mobile phase (chloroform) was 1 mL min^-1^. Molecular weights were calibrated with polystyrene standards from Varian (Calibration Kit S-M2-10, Agilent Technologies, Palo Alto, CA, USA).

#### Melting temperature

Melting temperatures (T_m_), the apparent heat of fusion (ΔH_m_, the energy required to change a substance from the solid to the liquid state without changing its temperature), and the glass transition temperatures (T_g_, the temperature at which the transition in the amorphous regions between the glassy and rubbery state occurs) of PHAs were evaluated using TA Q2000 differential scanning calorimetry (DSC; TA Instruments, New Castle, DE, USA). Thermal data were collected under a nitrogen flow of 10 mL min^-1^. About 5 mg of melt-quenched PHA samples encapsulated in aluminum pans were heated from −40 to 200 °C at a rate of 10 °C min^−1^. The melting temperatures were determined from the position of the endothermic peaks. The apparent heat of fusion (ΔH_m_) was determined from the DSC endothermal peaks in the second scan. The glass transition temperature (T_g_) was taken as the inflection point of the specific heat increment at the glass–rubber transition.

#### Nuclear magnetic resonance (NMR)

^1^H-NMR spectra (400, 500, and 600 MHz) were recorded at room temperature, with shifts reported in parts per million downfield from tetramethylsilane and referenced to the residual solvent peak. ^13^C-NMR spectra (100 and 125 MHz, 1048 scans, delay time (d1) = 0.5 s) of PHAs were recorded at room temperature, with shifts reported in parts per million downfield from tetramethylsilane. PHA samples for NMR were prepared by adding approximately 3 mg of the PHA to 0.7 mL deuterated chloroform (CDCl_3_), with gentle heating until the PHA had dissolved.

#### Preparations of PHA thin films

In order to produce solution-cast films ~150 µm thick, 0.4 g of bioplastic powder was added to 20 mL of chloroform (ACS reagent grade; ACROS Organics, Morris Plains, NJ, USA). The chloroform and bioplastic were stirred while being heated at the boiling point of chloroform (61 °C) for 2 h, until the bioplastic was completely dissolved. A reflux condenser was used to prevent excessive evaporation of the chloroform. After full dissolution, the liquid was poured into a 60 mm diameter glass petri dish and covered to allow the chloroform to slowly evaporate over 24 h. As noted by Bergstrand (2012), drying rate is a critical parameter for attaining homogeneous films. The optimal evaporation rate was observed when the gap between the lid and the place was between 0.3 and 0.6 mm. Dried films of all bioplastics easily separated from the glass dish and were further trimmed to specimen sizes appropriate for testing. Plastic films were stored at −15 °C until testing to minimize aging effects (Srubar et al. 2012).

#### Tensile properties

Young’s modulus (E, a measure of stiffness of an elastic material), tensile strength (σ_t_, the resistance of a material to a force tending to tear it apart, measured as the maximum tension the material can withstand without tearing), and elongation at break (ε_b_; the ratio between changed length and initial length after breakage of the test specimen) were determined using an Instron 5565 Universal Testing Machine (Instron Corp., Canton, MA, USA). The dimensions of the specimens were 25 mm × 5 mm × 0.1 mm. The testing conditions used were: cross head speed of 5 mm min^-1^ and load cell of 0.1 kN.

#### Statistical sequence analysis of P(3HB-*co*-4HB)

The method used is described by Doi et al. (1990). ^13^C-NMR spectroscopy (125 MHz, 6800 scans, delay time (d1) = 5 s, room temperature) was conducted on a P(3HB-*co*-4HB) sample (Additional file 1: Figure S10) with the fraction of 3HB units, F_(3HB)_, equal to 0.9141 and fraction of 4HB units, F_(4HB)_, equal to 0.0859 (obtained from integration of ^1^H-NMR spectra). For a statistically random copolymer, Bernoullian statistics can be applied to calculate the expected fractions of diad sequences F_(3HB)(3HB)_, F_(3HB)(4HB)_, F_(4HB)(3HB)_, and F_(4HB)(4HB)_: F_(3HB)(3HB)_ = F_(3HB)_^2^, F_(3HB)(4HB)_ = F_(4HB)(3HB)_ = F_(3HB)_(1–F_(3HB)_), and F_(4HB)(4HB)_ = F_(4HB)_^2^. Observed diad, triad and tetrad fractions (via peak integration on the ^13^C-NMR spectrum) were compared to the calculated diad, triad and tetrad fractions for a statistically random copolymer with F_(3HB)_ = 0.9141 and F_(4HB)_ = 0.0859. Finally, the parameter D, which describes the randomness of the polymer chain (with D = 1.0 for a statistically random copolymer), was calculated using the equation D = F_(3HB)(3HB)_F_(4HB)(4HB)_/F_(3HB)(4HB)_F_(4HB)(3HB)_.

## Results

### Production of two/three component copolymers using various organic acid co-substrates

**Fig. 1:**
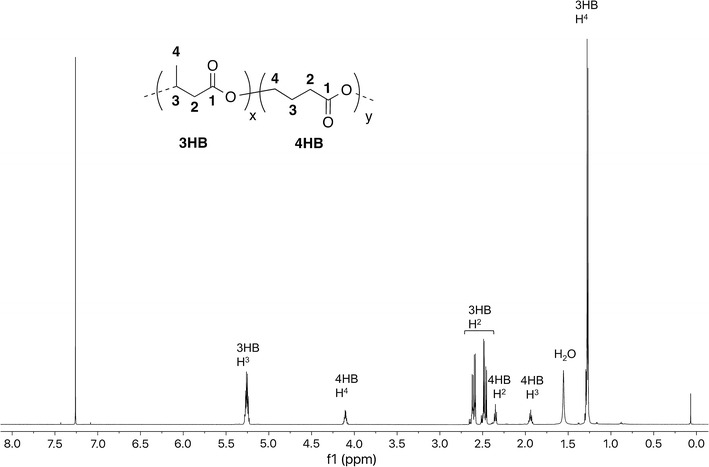
^1^H-NMR spectrum of P(3HB-*co*-4HB) produced by *M. parvus* OBBP

**Fig. 2:**
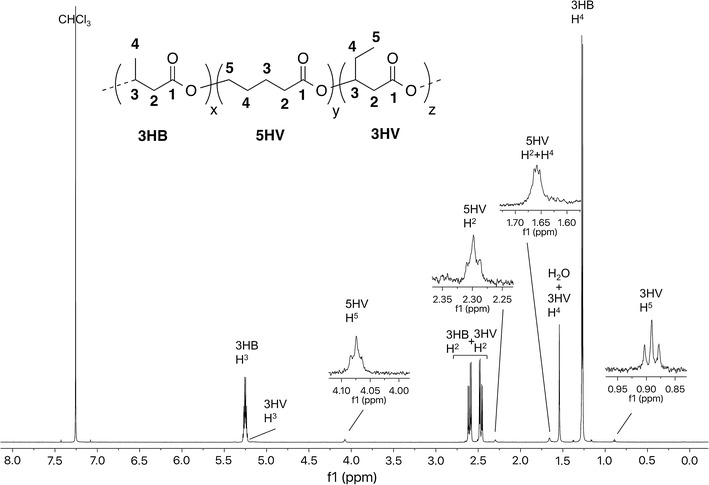
^1^H-NMR spectrum of P(3HB-*co*-5HV-*co*-3HV) produced by *M. parvus* OBBP

**Fig. 3:**
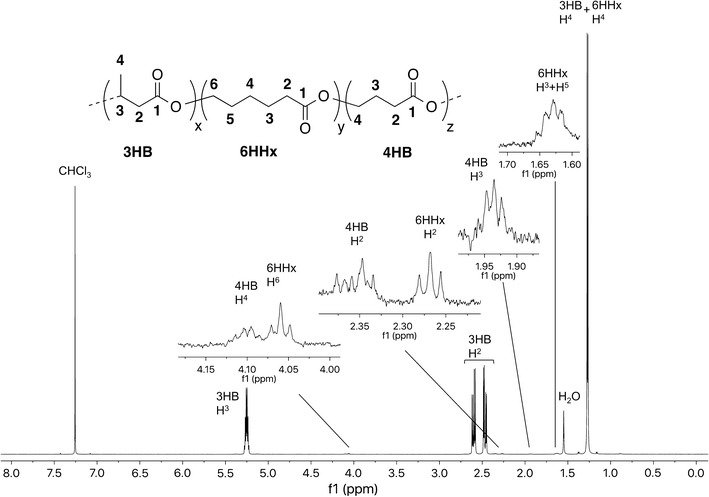
^1^H-NMR spectrum of P(3HB-*co*-6HHx-*co*-4HB) produced by *M. parvus* OBBP

Table 1 summarizes PHA copolymer production results for *M. parvus* OBBP. Integration of ^1^H-NMR spectra was used to determine monomer compositions. When grown with CH_4_ alone (Additional file 1: Figure S4), P3HB (42 ± 3 wt%) was the sole product. When 1.2 mM of butyrate (Additional file 1: Figure S5) or 3HB (Additional file 1: Figure S6) was added to harvested cells (without nitrogen in the incubation medium), P3HB was again the sole product. The P3HB content ranged from 55 ± 3 to 59 ± 5 wt%. When 1.2 mM of 4HB was added as co-substrate (Fig. 1), 4HB units were incorporated. The wt% P(3HB-*co*-4HB) was 50 ± 4 wt%, and the mol% 4HB was 9.5 mol%. When 1.2 mM of valerate was added as co-substrate (Additional file 1: Figure S7), 3HV units were incorporated. The wt% PHBV was 54 ± 4 wt%, and the mol% 3HV was 25.0 mol%. When 1.2 mM of 5HV was added as a co-substrate (Fig. 2), 5HV and 3HV units were incorporated, forming a three-component PHA. The mol% 5HV was 3.6 mol% and the mol% 3HV was 1.5 mol%. When 1.2 mM of hexanoate was added (Additional file 1: Figure S8), P3HB was the sole product. The wt% P3HB was 56 ± 4 wt%. When 1.2 mM of 6HHx was added as a co-substrate (Fig. 3), 6HHx and 4HB units were incorporated, forming a three-component PHA. The mol% 6HHx was 1.4 mol% and the mol% 4HB was 1.0 mol%. When 1.2 mM of octanoate was added (Additional file 1: Figure S9), P3HB was the sole product. The wt% P3HB was 54 ± 3 wt%. In all cases, the primary substrate was CH_4_. In the absence of CH_4_, no PHA was synthesized, an observation consistent with our previous observation that CH_4_ oxidation is required for methanotrophic PHA synthesis (Myung et al. 2016a).

**Table 1:** PHA production using various fatty acid co-substrates by *M. parvus* OBBP

Co-substrates	wt% PHA polymer	PHA monomer ratio (mol%)					TSS (mg/L)
		3HB	3HV	4HB	5HV	6HHx	
None	42 ± 3	100	0	0	0	0	1600 ± 180
Butyrate (1.2 mM)	55 ± 3	100	0	0	0	0	1660 ± 200
3-Hydroxybutyrate (1.2 mM)	59 ± 5	100	0	0	0	0	1820 ± 220
4-Hydroxybutyrate (1.2 mM)	50 ± 4	91.5	0	9.5	0	0	1720 ± 240
Valerate (1.2 mM)	54 ± 4	75.0	25.0	0	0	0	1760 ± 160
5-Hydroxyvalerate (1.2 mM)	48 ± 4	95.0	1.4	0	3.6	0	1640 ± 180
Hexanoate (1.2 mM)	56 ± 4	100	0	0	0	0	1740 ± 200
6-Hydroxyhexanoate (1.2 mM)	48 ± 3	97.6	0	1.0	0	1.4	1680 ± 220
Octanoate (1.2 mM)	54 ± 3	100	0	0	0	0	1720 ± 180

### Influence of 4HB concentrations on P(3HB-*co*-4HB) production

**Fig. 4:**
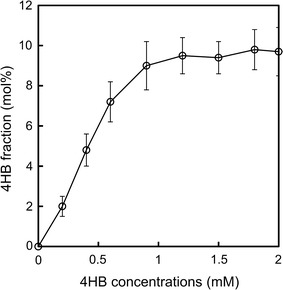
4HB fractions in P(3HB-*co*-4HB) produced relative to initial 4HB concentrations. The primary carbon substrate is CH_4_. The *errors bars* represent standard deviations for triplicate batch cultures

To understand the effect of co-substrate on product formation, a range of 4HB concentrations were added, and the mol% 4HB in P(3HB-*co*-4HB) was monitored (Fig. 4). For added 4HB levels <1 mM, the mol% 4HB of the PHA copolymer increased with increasing 4HB concentrations. At higher levels, the 4HB fraction stabilized at ~10 mol%.

### Molecular weight characterization

Table 2 illustrates the number average molecular weights (M_n_) and the molecular weight distributions (M_w_/M_n_; a measure of the distribution of molecular mass in a given polymer sample) of PHAs produced by *M. parvus* OBBP. The M_n_ of all PHAs tested were above 1.0 × 10^6^ Da, indicating production of PHAs with high molecular weights. These values are comparable to those of heterotrophic enrichments and to commercial P3HB and PHBV powders (Table 2, Sigma-Aldrich, St Louis, MO, USA) (Myung et al. 2014), but are more uniform, with higher M_n_ and lower M_w_/M_n_.

**Table 2:** Physical properties of the methanotroph produced PHA polymers

PHA products	Molecular weights		Thermal properties			Mechanical properties		
	M_n_	M_w_/M_n_	T_m_ (°C)	ΔH_m_ (J/g)	T_g_ (°C)	E (GPa)	σ_t_ (MPa)	ε_b_ (%)
P3HB	1.48 E+06	1.82	178	83	3	3.0	43.2	5.2
P(3HB-*co*-24 mol% 3HV)	1.32 E+06	2.24	147	45	−1	1.0	22.0	50.5
P(3HB-*co*-3.0 mol% 4HB)	1.33 E+06	2.12	148	65	−2	1.2	35.6	176
P(3HB-*co*-9.5 mol% 4HB)	1.22 E+06	2.01	135	47	−5	0.8	31.2	284
P(3HB-*co*-3.6 mol% 5HV-*co*-1.4 mol% 3HV)	1.26 E+06	2.17	144	44	−2	0.8	29.9	106
P(3HB-*co*-1.4 mol% 6HHx-*co*-1.0 mol% 4HB)	1.27 E+06	2.11	150	40	−1	0.7	27.6	134
Commercial P3HB	7.38 E+05	2.02						
Commercial PHBV	4.48 E+05	2.18						

*M*_*n*_ number average molecular weight, *M*_*w*_ weight average molecular weight, *T*_*m*_ melting temperature, *ΔH*_*m*_ apparent heat of fusion, *T*_*g*_ glass transition temperature, *E* Young’s modulus, *σ*_*t*_ tensile strength, *ε*_*b*_ elongation at break

### Melting temperatures of the PHA generated

The thermal behaviors of the two/three component PHA copolymers were investigated using differential scanning calorimetry (DSC) (Table 2). The melting temperature (T_m_) of P3HB was highest at 178 °C and were lower for the two/three component PHA copolymers tested, ranging from 135 to 148 °C. The apparent heat of fusion (ΔH_m_) was highest at 83 J/g and were the two/three component PHA copolymers tested, ranging from 44 J/g to 65 J/g. The glass transition temperature (T_g_) of P3HB was highest at 3 °C and were lower for the two/three component PHA copolymers tested, ranging from −5 to −1 °C.

### Mechanical properties of the produced PHA copolymers

Table 2 illustrates the mechanical properties of thin-film PHA samples determined using Instron Universal Testing Machine. P3HB had the highest Young’s modulus (E) of 3.0 GPa, and the other PHA copolymers had significantly lower Young’s modulus ranging from 0.7 to 1.2 GPa. The tensile strength (σ_t_) was also highest for P3HB at 43.2 MPa, and the tensile strengths of other PHA copolymers tested ranged from 22.0 to 35.6 MPa. However, elongation at break (ε_b_) was greatly improved from 5.2% to 50.5–284% upon copolymerization.

### Statistical sequence analysis of P(3HB-*co*-4HB)

The observed diad fractions of the P(3HB-*co*-4HB) sample (F_(3HB)_ = 0.9141 and F_(4HB)_ = 0.0859) match closely with calculated diad fractions based on a statistically random copolymer obeying Bernoullian statistics (Additional file 1: Table S1). The value of the parameter D, calculated using the carbonyl peaks of the ^13^C-NMR spectrum, was 1.29. This is close to the value of D for a statistically random copolymer (D = 1.0).

## Discussion

Our group and others have previously reported production of P3HB and PHBV by pure culture methanotrophs using CH_4_ and various co-substrates (Cal et al. 2016; Flanagan et al. 2016; Myung et al. 2016a). The general rule was that even-numbered carbon co-substrates (e.g. 3HB, crotonate) led to production of P3HB, whereas odd-numbered carbon co-substrates (e.g. propionate, valerate, 2-pentenoate, or pentanol) led to production of PHBV. Depending on the number of carbon atoms, fatty acid substrates are processed via the beta-oxidation pathway into either acetyl-CoA or propionyl-CoA, the precursors of P3HB and PHBV.

Addition of ω-hydroxyalkanoate co-substrates resulted in a different outcome. In this case, *M. parvus* OBBP synthesized a random copolymer containing 3HB and ω-hydroxyalkanoate monomers. While various species of bacteria have synthesized PHAs containing 4HB or 5HV monomers (Poirier et al. 1995; Chanprateep et al. 2010; Chuah et al. 2013), this is the first report of methane-dependent production of PHA copolymers other than PHBV.

**Fig. 5:**
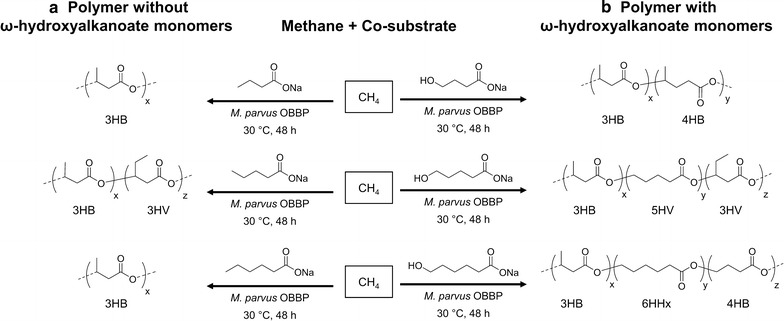
Scheme of PHA production using methane and co-substrates. **a** Without the hydroxyl group and **b** with the hydroxyl group

**Fig. 6:**
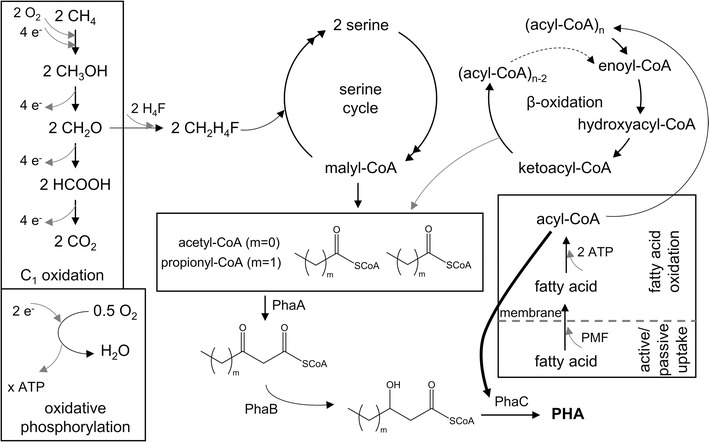
C_1_-oxidation dependent synthesis of PHAs in serine-cycle methanotrophic bacteria. The *bold arrow* denotes an acyl-CoA pathway likely activated by ω-hydroxyalkanoate co-substrates

The outcome of ω-hydroxyalkanoate co-substrate addition was dependent upon the presence and position of the hydroxyl group (Fig. 5; Table 1). When butyrate or 3HB was added, P3HB was produced; when 4HB was added, the resulting polymer was P(3HB-*co*-4HB). When valerate was added, the resulting polymer was PHBV, but when 5HV was added, the product was P(3HB-*co*-5HV-*co*-3HV). When hexanoate was added, the polymer product was P3HB, but when 6HHx was added, the product was P(3HB-*co*-6HHx-*co*-4HB). From these results, we can conclude that the presence of a hydroxyl group and its position play a key role in determining the composition of the PHA produced. When there is a hydroxyl group on the nth carbon (e.g. ω-hydroxyalkanoates), the co-substrate seems to be incorporated directly into the polymer by PHA synthase (PhaC) (Fig. 6), and it can also undergo beta-oxidation, as evidenced by formation of 4HB monomer units derived from the 6HHx co-substrate.

Thermal stability is critical for polymer melt processing. P3HB has a narrow processing window, melting at 175–180 °C and thermally degrading at ~190 °C. PHA copolymers produced by incorporation of ω-hydroxyalkanoate monomers have significantly lower melting temperatures (T_m_), expanding the processing window (Table 2). These copolymers also had lower T_g_ values suggesting increased chain mobility compared to P3HB. This would manifest as a decrease in brittleness, an expectation confirmed by the results of mechanical testing summarized below.

Key mechanical properties for useful application of bioplastics are Young’s modulus (E), tensile strength (σ_t_), and elongation at break (ε_b_). P3HB has high E and σ_t_, but is brittle, with a small ε_b_. Short chain-length monomers generally confer toughness and high crystallinity, and medium chain-length monomers generally confer elasticity and low crystallinity. Thus, a mixture of the two enables production of PHA that is both tough and elastic, an important combination of properties for many applications.

Heterotrophic bacteria have been shown to produce P(3HB-*co*-4HB) (Doi et al. 1990; Nakamura et al. 1992; Saito and Doi 1994; Chanprateep et al. 2010) and P(3HB-*co*-5HV-*co*-3HV) (Doi et al. 1987; Steinbuchel and Valentin 1995), but methanotrophic synthesis of these PHA copolymers has not been reported previously. To the best of our knowledge, this is the first reported microbial synthesis of P(3HB-*co*-6HHx-*co*-4HB). Generalizing these results, we envision that control over the structure and concentration of added co-substrates will enable synthesis of copolymers suitable for a broad range of applications (Fig. 4).

Use of CH_4_ as the primary substrate for PHA synthesis is of interest because CH_4_ is abundant, cheap, and its use does not adversely impact the food supply, unlike cultivated feedstock (Levett et al. 2016). The cost of ω-hydroxyalkanoate co-substrates is high, but can be significantly reduced when lactones are used as the precursor for chemical synthesis of ω-hydroxyalkanoates (see “Materials and methods” section). We conclude that provision of methane as primary substrate and addition of ω-hydroxyalkanoate as co-substrates is a promising route for synthesis of polymers with tunable physical properties.

## Additional file

**Additional file 1.** Additional tables and figures.

## Abbreviations

PHA: polyhydroxyalkanoates
P(3HB-*co*-4HB): poly(3-hydroxybutyrate-*co*-4-hydroxybutyrate)
P(3HB-*co*-5HV-*co*-3HV): poly(3-hydroxybutyrate-*co*-5-hydroxyvalerate-*co*-3-hydroxyvalerate)
P(3HB-*co*-6HHx-*co*-4HB): poly(3-hydroxybutyrate-*co*-6-hydroxyhexanoate-*co*-4-hydroxybutyrate)
4HB: 4-hydroxybutyrate
3HB: 3-hydroxybutyrate
5HV: 5-hydroxyvalerate
6HHx: 6-hydroxyhexanoate
P3HB: poly(3-hydroxybutyrate
PHBV: poly(3-hydroxybutyrate-*co*-3-hydroxyvalerate
3HV: 3-hydroxyvalerate
AMS: ammonium mineral salts
TSS: total suspended solids
GPC: gel permeation chromatography
NMR: nuclear magnetic resonance
