# Supplementary material for: Expanding the range of polyhydroxyalkanoates synthesized by methanotrophic bacteria through the utilization of omega-hydroxyalkanoate co-substrates
Source: AMB Express. 2017 Jun 5;7:118. doi: 10.1186/s13568-017-0417-y (PMC5459778; doi:10.1186/s13568-017-0417-y)

AMB Express

Expanding the range of polyhydroxyalkanoates synthesized by methanotrophic bacteria through the utilization of omega-hydroxyalkanoate co-substrates

Jaewook Myung, James C. A. Flanagan, Robert M. Waymouth, Craig S. Criddle*

* Correspondence to: J. Myung

Email: jjaimyung@psu.edu

Phone: (+1) 814-865-4276

SUPPORTING INFORMATION

Contents:

Statistical sequence analysis of P(3HB-*co*-4HB) (**Table S1)**

^{1}H- and ^{13}C-NMR spectra of synthesized ω-hydroxyalkanoates (**Fig. S1–S3**)

^{1}H-NMR spectra of synthesized polyhydroxyalkanoates (**Fig. S4–S9**)

^{1}H- and ^{13}C-NMR of the P(3HB-*co*-4HB) used for statistical sequence analysis (**Fig. S10**)

Table S1 Statistical sequence analysis of P(3HB-*co*-4HB).

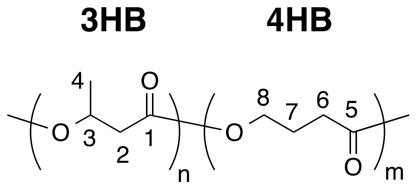

Peak	Chemical shift (ppm)	Sequence (* denotes peak assignment, with adjacent repeating units listed before and/or after)	Normalized peak integration (from ^{13}C-NMR)	
			observed from ^{13}C-NMR	calculated, based on $F_{(3HB)}$= 0.9141 $F_{(4HB)}$= 0.0859 from ^{1}H-NMR
CH_3 (4)	19.77	3HB-3HB*-3HB	0.85	0.84
	19.82	3HB-3HB*-4HB	0.08	0.08
	19.87	4HB-3HB*-3HB	0.07	0.08
	19.93	4HB-3HB*-4HB	<0.01	0.01
CH_2 (7)	23.95	3HB-4HB*	>0.99	0.91
	24.00	4HB-4HB*	<0.01	0.08
CH_2 (6)	30.59	3HB-4HB*-4HB	0.01	0.08
	30.66	4HB-4HB*-4HB	<0.01	0.01
	30.79	3HB-4HB*-3HB-3HB	0.78	0.76
	30.82	3HB-4HB*-3HB-4HB	0.15	0.07
	30.85	4HB-4HB*-3HB-3HB	0.07	0.07
	30.88	4HB-4HB*-3HB-4HB	<0.01	0.01
CH_2 (2)	40.54	3HB-3HB*-4HB	0.08	0.08
	40.65	4HB-3HB*-4HB	0.04	0.01
	40.80	3HB-3HB*-3HB-3HB	0.72	0.77
	40.86	3HB-3HB*-3HB-4HB	0.09	0.07
	40.90	4HB-3HB*-3HB-3HB	0.06	0.07
	40.95	4HB-3HB*-3HB-4HB	0.01	<0.01
CH_2 (8)	63.54	4HB-4HB*	<0.01	0.08
	63.64	3HB-4HB*	>0.99	0.91
CH (3)	67.42	4HB-3HB*	0.10	0.08
	67.62	3HB-3HB*	0.90	0.91
CO (1)	169.15	3HB-3HB*-3HB	0.79	0.76
	169.25	4HB-3HB*-3HB	0.06	0.07
	170.00	3HB-3HB*-4HB	0.06	0.07
	170.08	4HB-3HB*-4HB	0.01	0.01
CO (5)	171.85	4HB-3HB*	0.08	0.08
	171.88	4HB-3HB*		
	171.92	4HB-3HB*		
	172.62	3HB-4HB*-4HB	<0.01	0.01
	172.67	4HB-4HB*-4HB	<0.01	<0.01

This method is described by Doi. et al. (1990).

$D = F_{(3HB)(3HB)}F_{(4HB)(4HB)}/F_{(3HB)(4HB)}F_{(4HB)(3HB)}$ = [(0.76+0.07)*(0.01)]/[(0.08)*(0.08)] = 1.29

Fig. S1 (a) ^{1}H- and (b) ^{13}C-NMR spectra of synthesized 4-hydroxybutyrate (4HB).

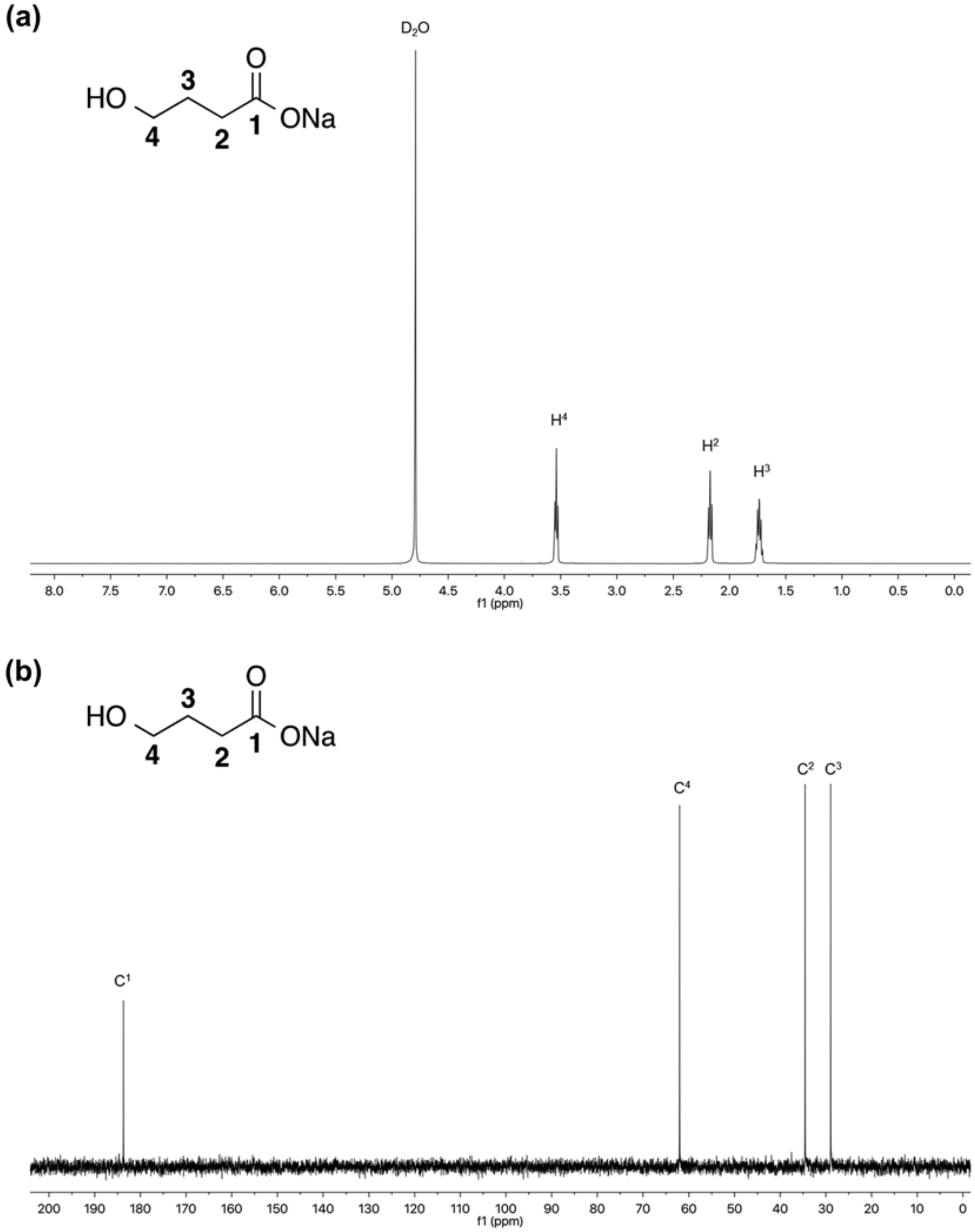

Fig. S2 (a) ^{1}H- and (b) ^{13}C-NMR spectra of synthesized 5-hydroxyvalerate (5HV).

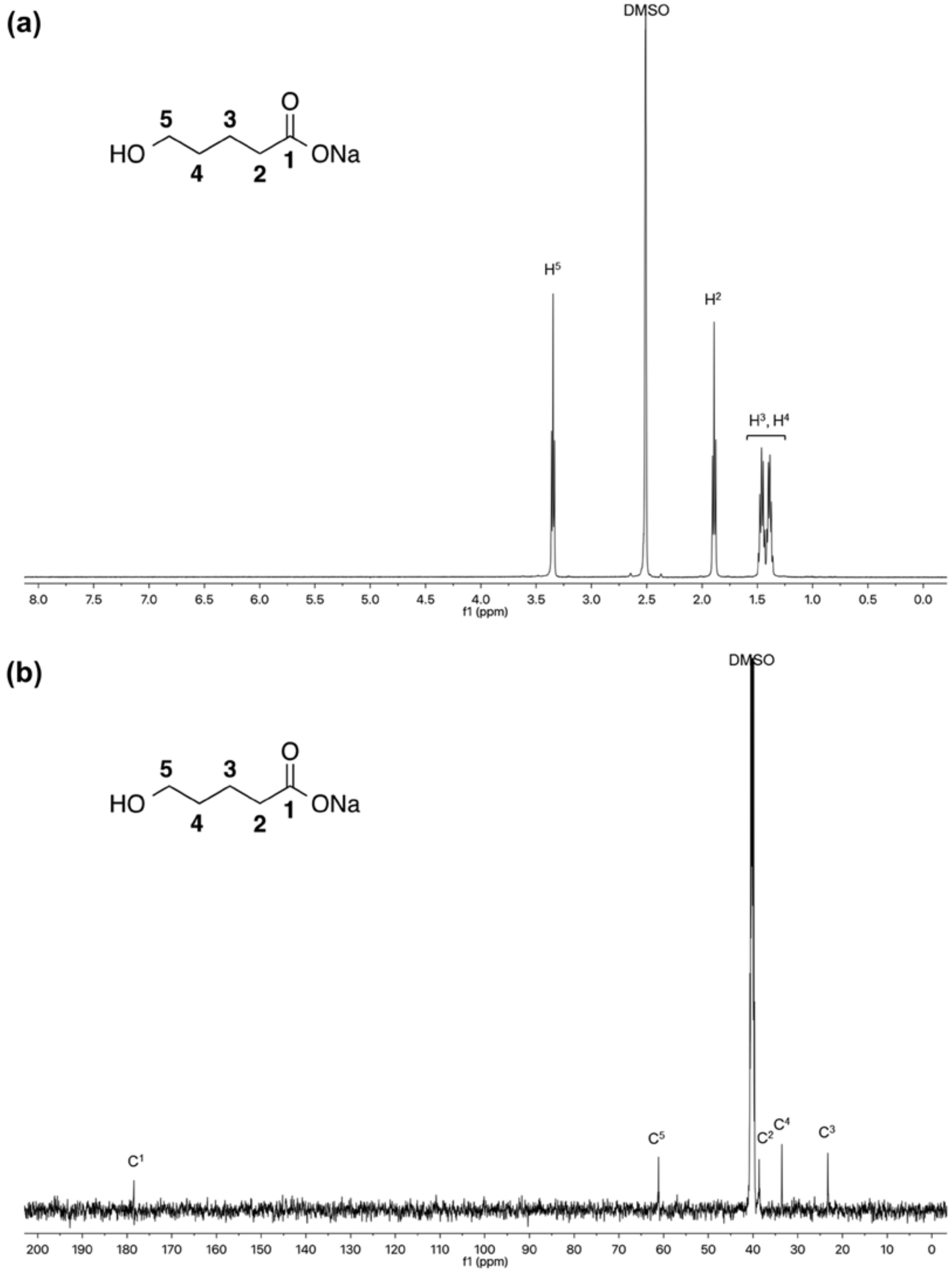

Fig. S3 (a) ^{1}H- and (b) ^{13}C-NMR spectra of synthesized 6-hydroxyhexanoate (6HHx).

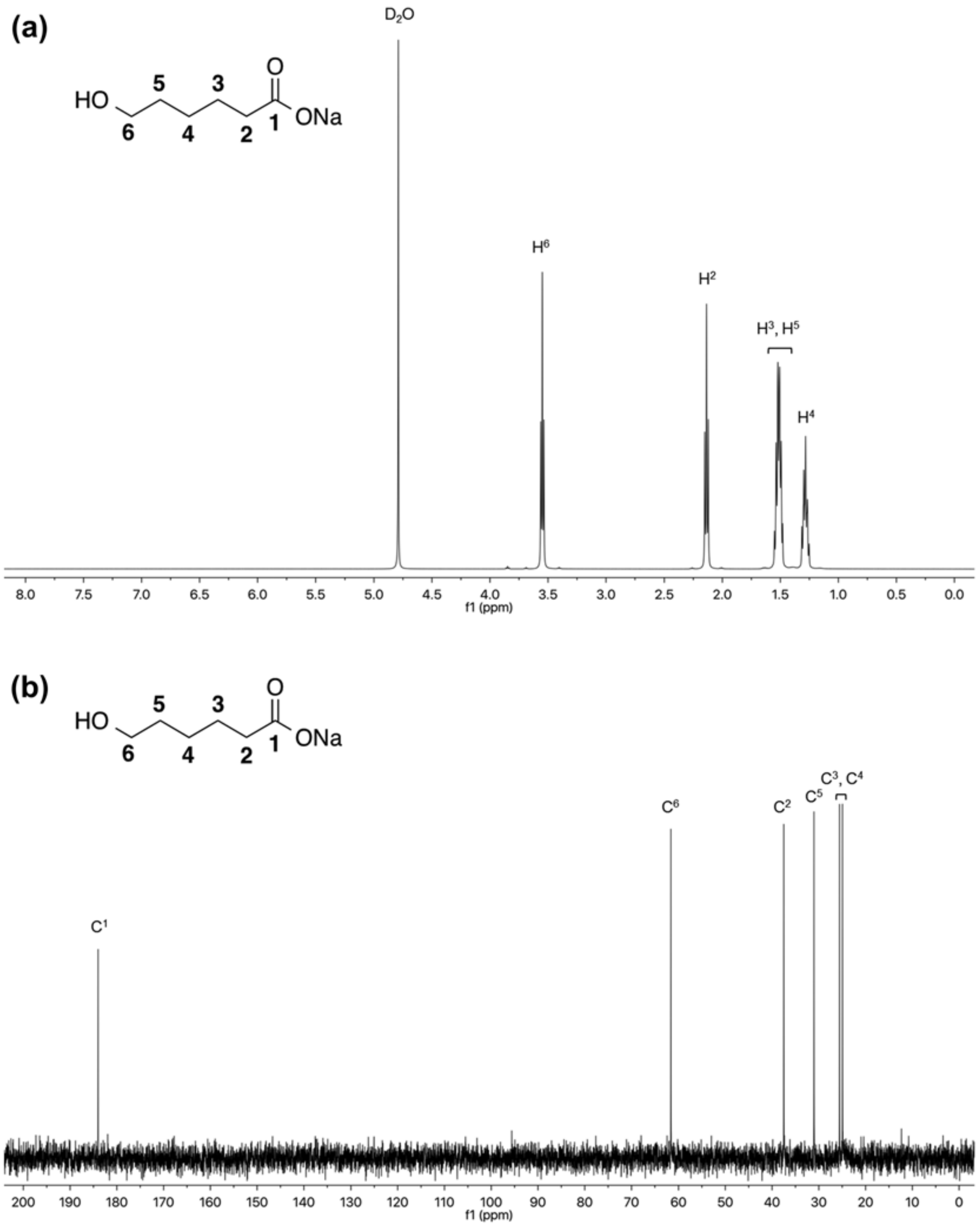

Fig. S4 ^{1}H-NMR spectra of synthesized polyhydroxyalkanoates when no co-substrate was added.

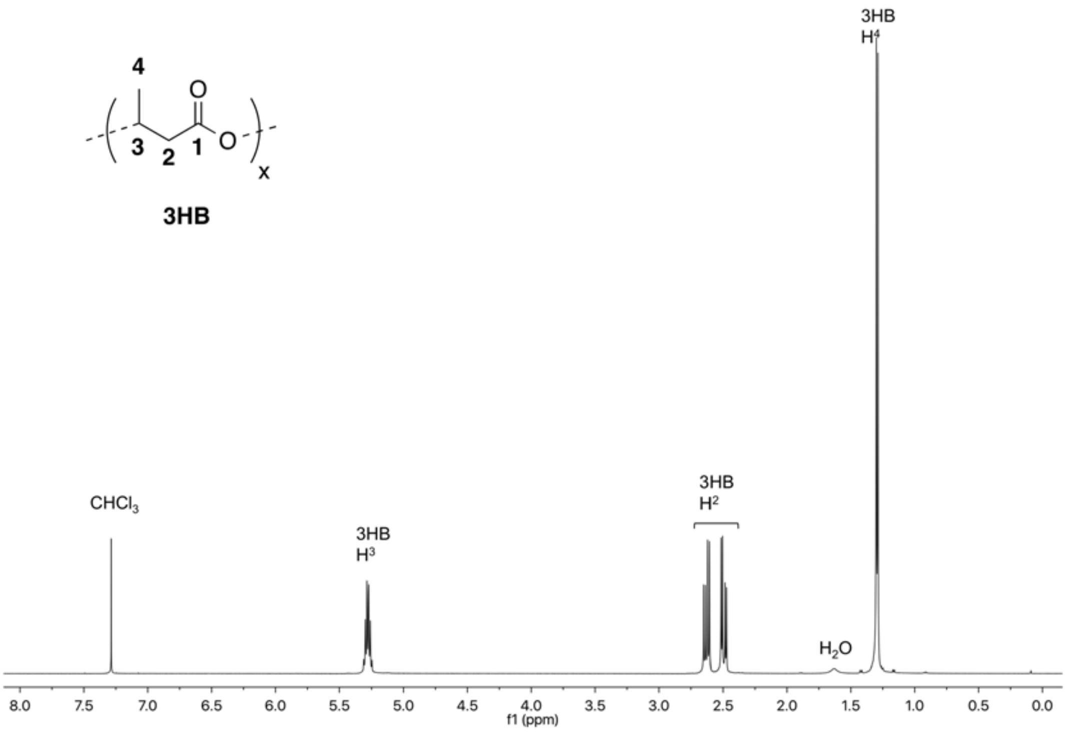

Fig. S5 ^{1}H-NMR spectra of synthesized polyhydroxyalkanoates when butyrate was added as a co-substrate.

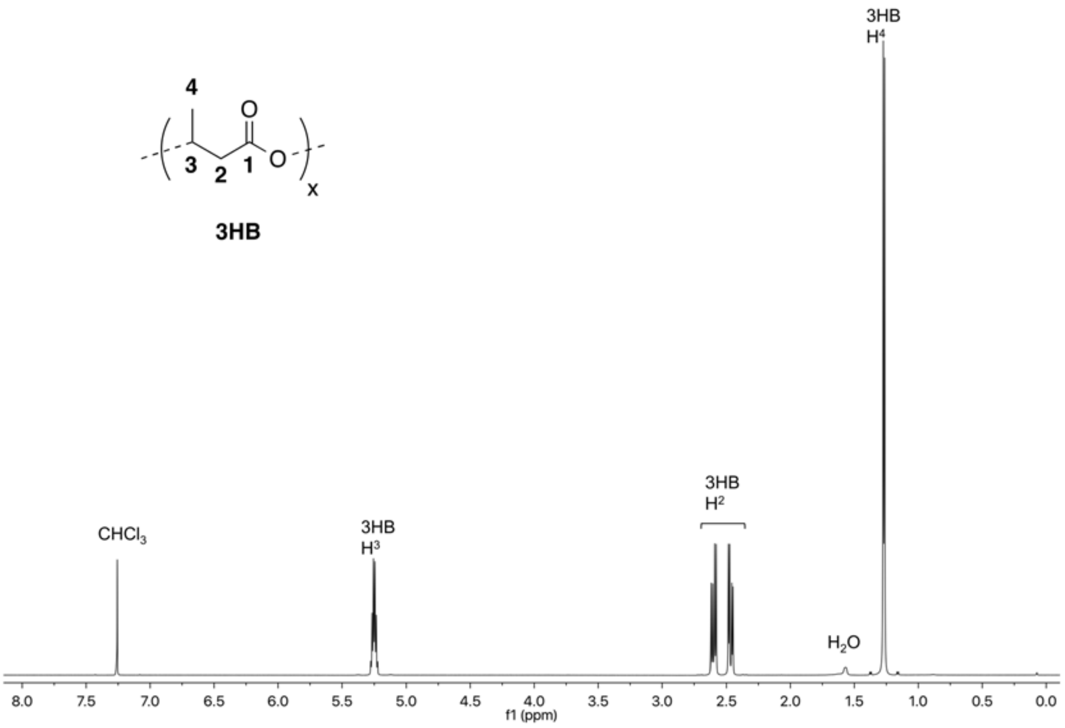

Fig. S6 ^{1}H-NMR spectra of synthesized polyhydroxyalkanoates when 3-hydroxybutyrate (3HB) was added as a co-substrate.

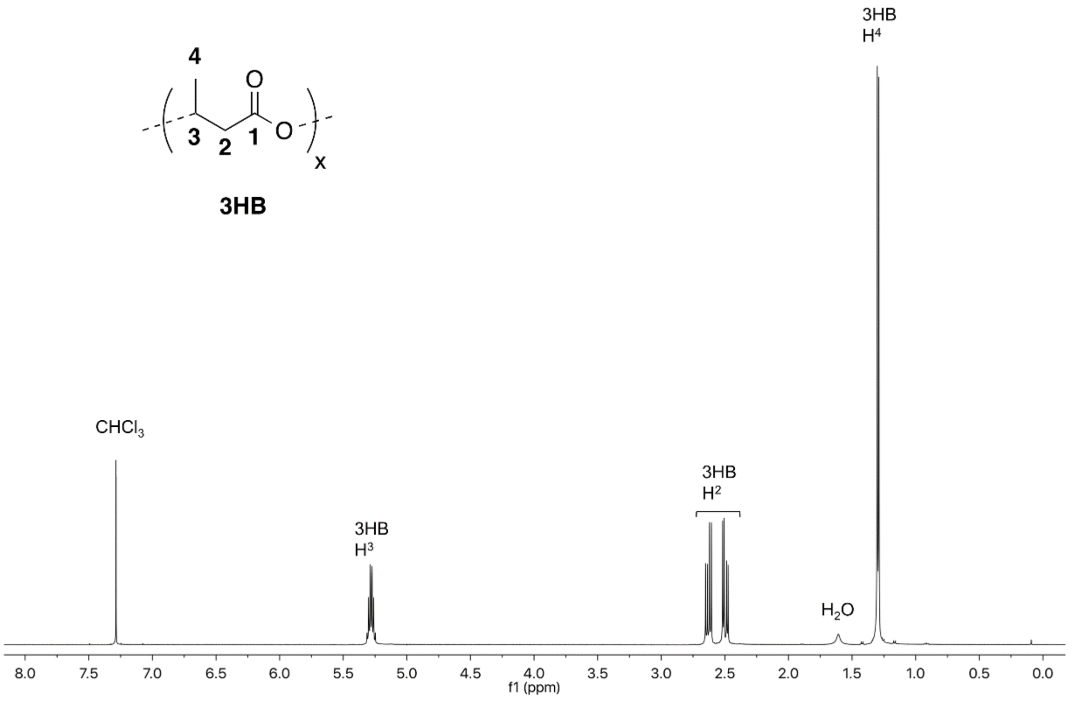

Fig. S7 ^{1}H-NMR spectra of synthesized polyhydroxyalkanoates when valerate was added as a co-substrate.

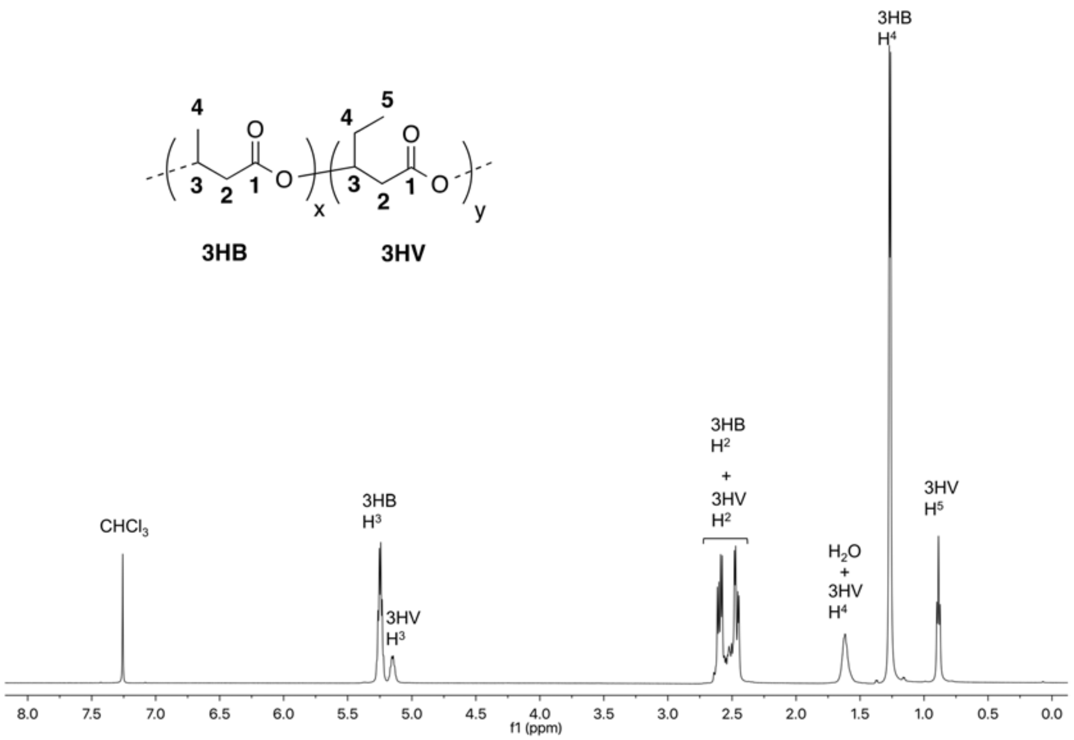

Fig. S8 ^{1}H-NMR spectra of synthesized polyhydroxyalkanoates when hexanoate was added as a co-substrate.

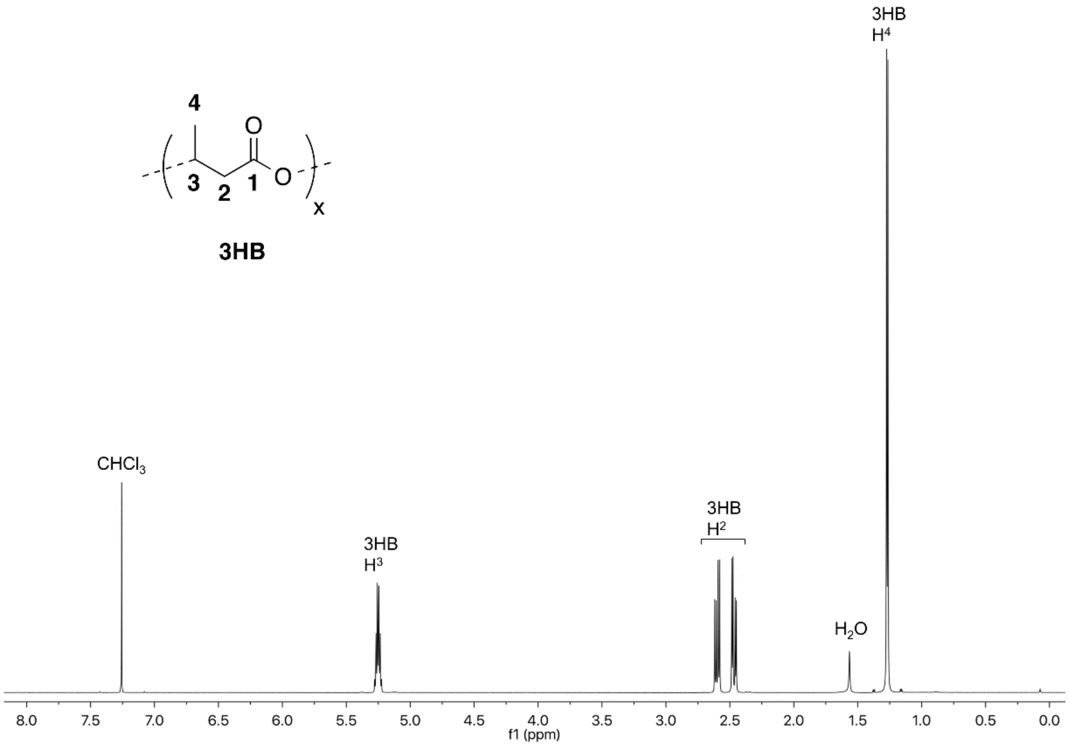

Fig. S9 ^{1}H-NMR spectra of synthesized polyhydroxyalkanoates when octanoate was added as a co-substrate.

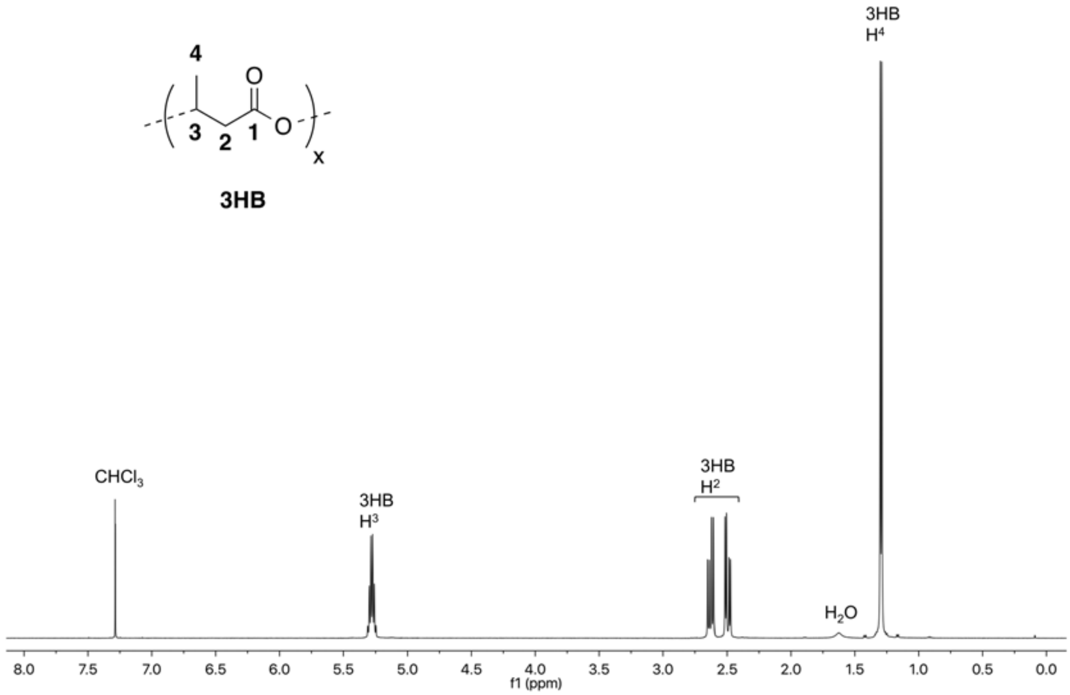

Fig. S10 (a) ^{1}H- and (b) ^{13}C-NMR of the P(3HB-*co*-4HB) used for statistical sequence analysis

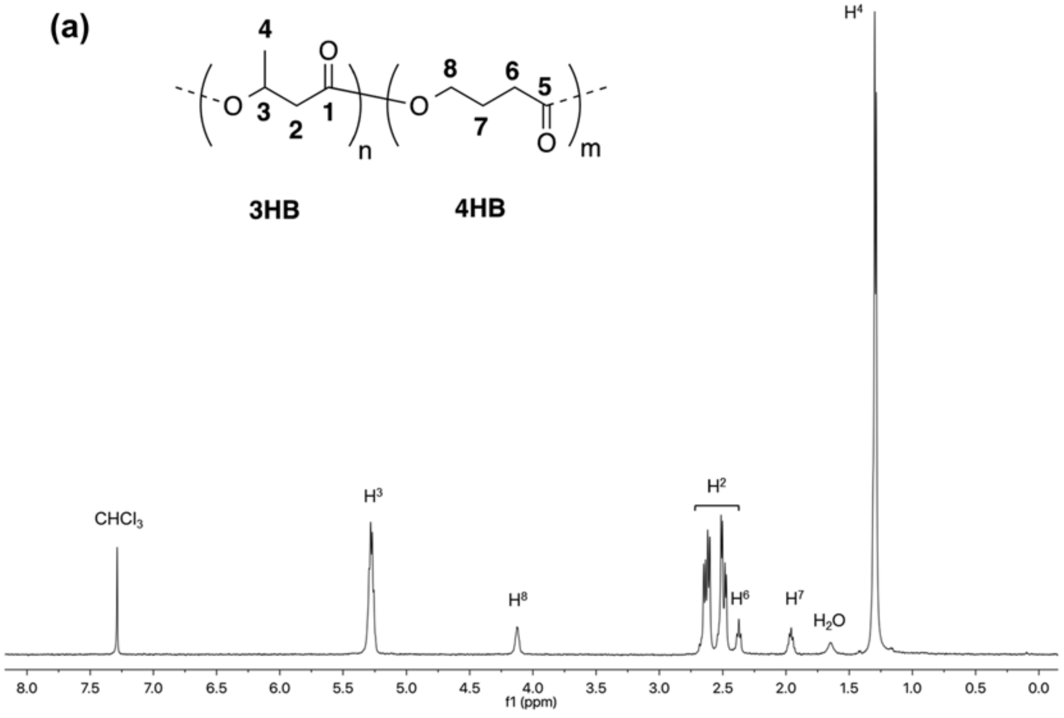

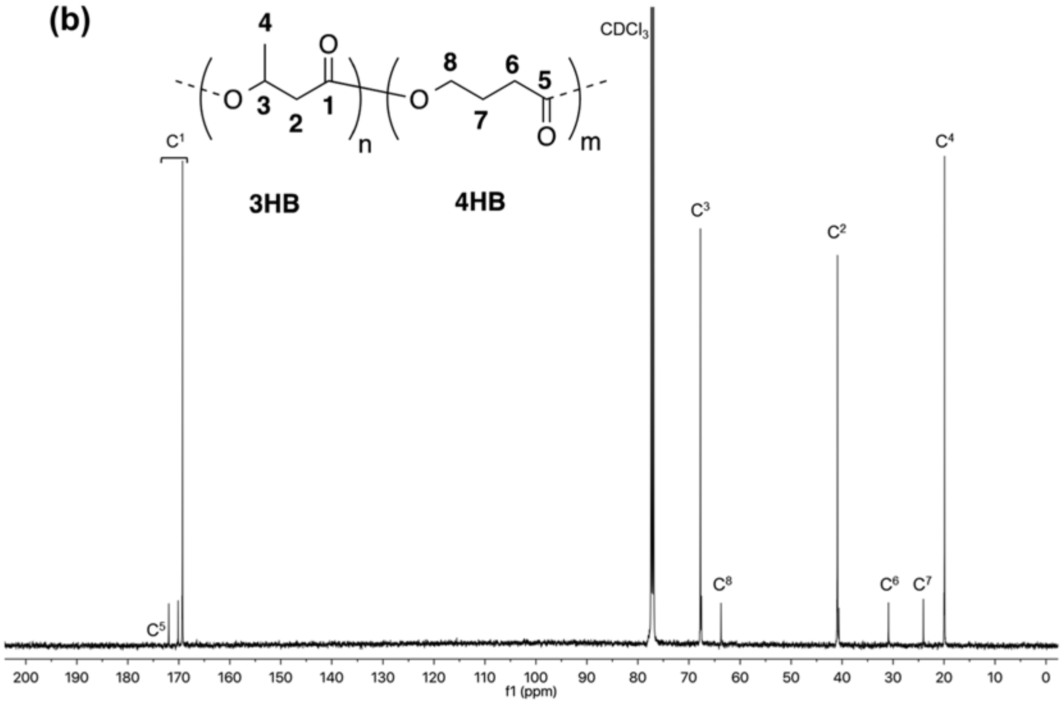

Supplement: Supplementary file 1 — Additional file 1. Additional tables and figures. [file 13568_2017_417_MOESM1_ESM.pdf]
